# Isolated septic non-otogenic lateral sinus thrombosis complicating nasopharyngitis in a 2-month-old infant: a case report

**DOI:** 10.1259/bjrcr.20170053

**Published:** 2017-06-20

**Authors:** Peter Waweru, Alice Wakonyo, Hussein Dossajee, Bhupi Reel

**Affiliations:** ^1^Department of Surgery, MP Shah Hospital, Nairobi, Kenya; ^2^Department of Pediatrics and Child Health, MP Shah Hospital, Nairobi, Kenya

## Abstract

Nasopharyngeal infections are among the commonest diagnosed infections in infants. Largely treated supportively, these infections are considered harmless. These can however lead to serious complications from local spread and septicemia. With local extension, inflammatory neck masses (abscesses) can swiftly lead to life-threatening complications including mediastinitis, airway compromise and sinovenous thrombosis. Here, we report a 2-month-old infant with initial rhinopharyngitis and subsequent extensive deep neck abscesses with consequent dural sinus thrombosis successfully managed with antibiotics, anticoagulants and drainage. While such cases have been presented before, it is the pathophysiology and extent of sinus thrombosis in our case that is eccentric.

## Background

The deep neck spaces, lined by *fascia cervicalis profunda,* include the parapharyngeal, retropharyngeal and prevertebral spaces. Here, common infections including upper respiratory tract, dental and ear infections may spread from lymphadenitis to cellulitis, phlegmon and eventually to abscesses.^1^ These deep neck infections (DNIs) often begin rapidly and can lead to life-threatening complications, including airway obstruction, mediastinitis, pericarditis, arterial erosion and jugular vein thrombosis.^2^ While not the most dreaded, it is the rarity and associated morbidity of sinovenous thrombosis that makes it eccentric, and thus the subject of our case report. We report a case of a 2-month-old infant with rhinopharyngitis and subsequent extensive deep neck abscesses complicated by isolated lateral sinus thrombosis (LST). The unique etiology and pathophysiology of such non-otogenic isolated LST, as in our case, is discussed.

## Case report

A 2-month-old male infant was referred to our intensive care unit (ICU) from a peripheral facility where he had presented with one day of progressively worsening difficulty in breathing and feeding associated with multiple convulsions of generalized tonic clonic nature. On examination, he was dyspneic, afebrile and tachycardic, with cold extremities, weak pulses and a delayed capillary refill of 4 s. His blood pressure was 98/64 mmHg and blood sugar 14.4 mmol l^–1^. An arterial blood gas showed a mixed metabolic/respiratory acidosis with a pH of 7.1, hypercapnia (pCO_2_ 55 mmHg) and decreased bicarbonate levels (20 mEq l^–1^). He had a white cell count of 15.6 × 10^9^ µl^–1^, largely neutrophils (76%), a reactive thrombocytosis (605 × 10^9^ µl^–1^), an international normalized ratio (INR) 1.24 and a normal chest radiograph. Further history revealed that the infant had an uneventful natal and postnatal period, but had had 3 weeks of nasal congestion, treated with nasal saline drops, antihistamines, antibiotics and antipyretics.

In the ICU, he was immediately intubated (endotracheal tube 3.5 mm diameter, 11 cm length) and ventilated. He was put on broad spectrum antibiotics (ceftriaxone, clarithromycin) for severe pneumonia/sepsis, phenytoin and morphine.

**Figure 1.**
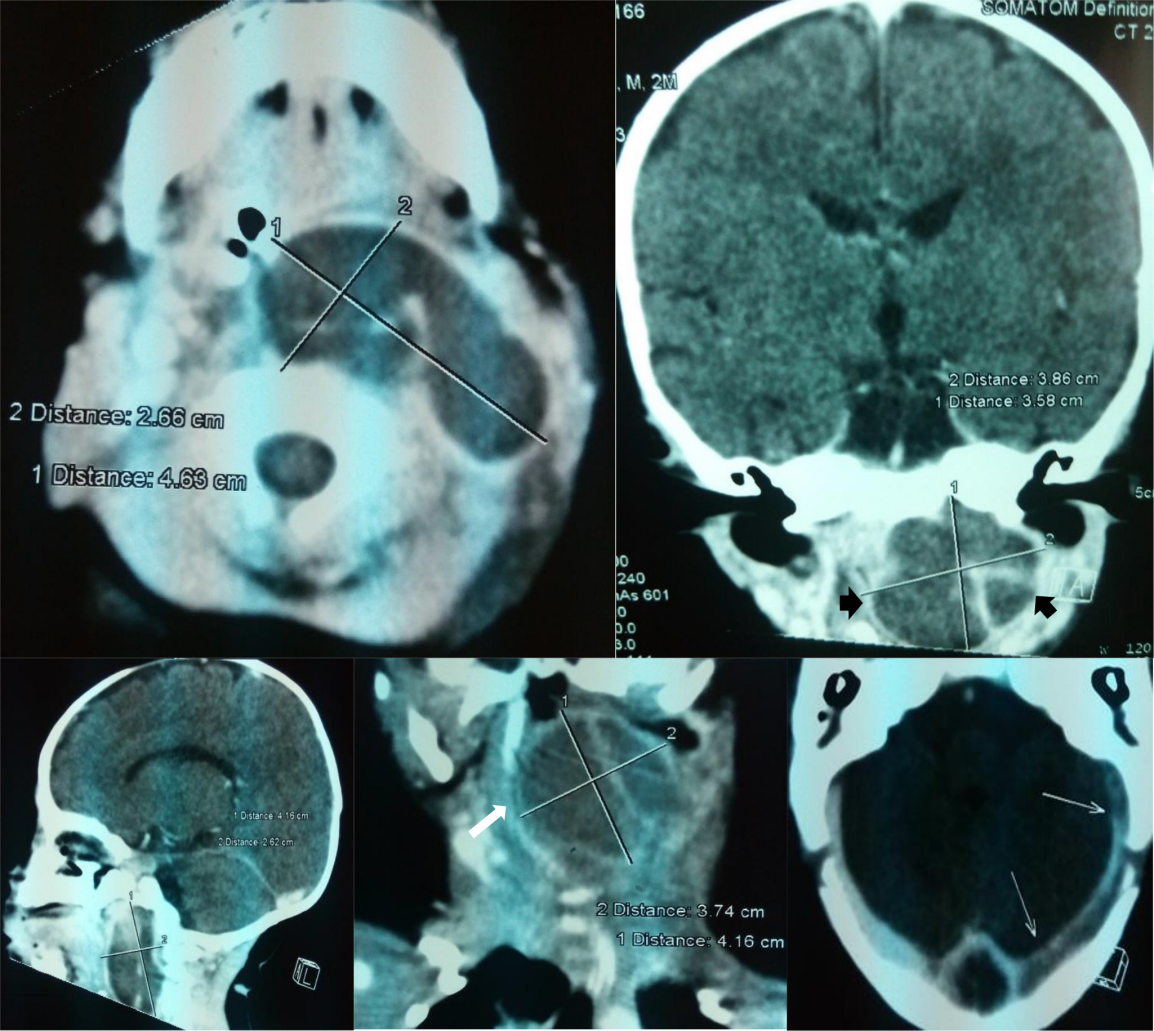
(a–e) Contrast enhanced CT scan images at the level of naso/oropharynx: axial (a), sagittal (c) and coronal (b) and (d) revealing an abscess within right retropharyngeal and parapharyngeal spaces seen as a low density lesion with thin rim of peripheral enhancement (black arrowheads on b). Also note the massive displacement of the trachea ((white arrow in d) and lateral sinus thrombosis on e) seen as filling defects in the sinus (white arrows).

**Figure 2.**
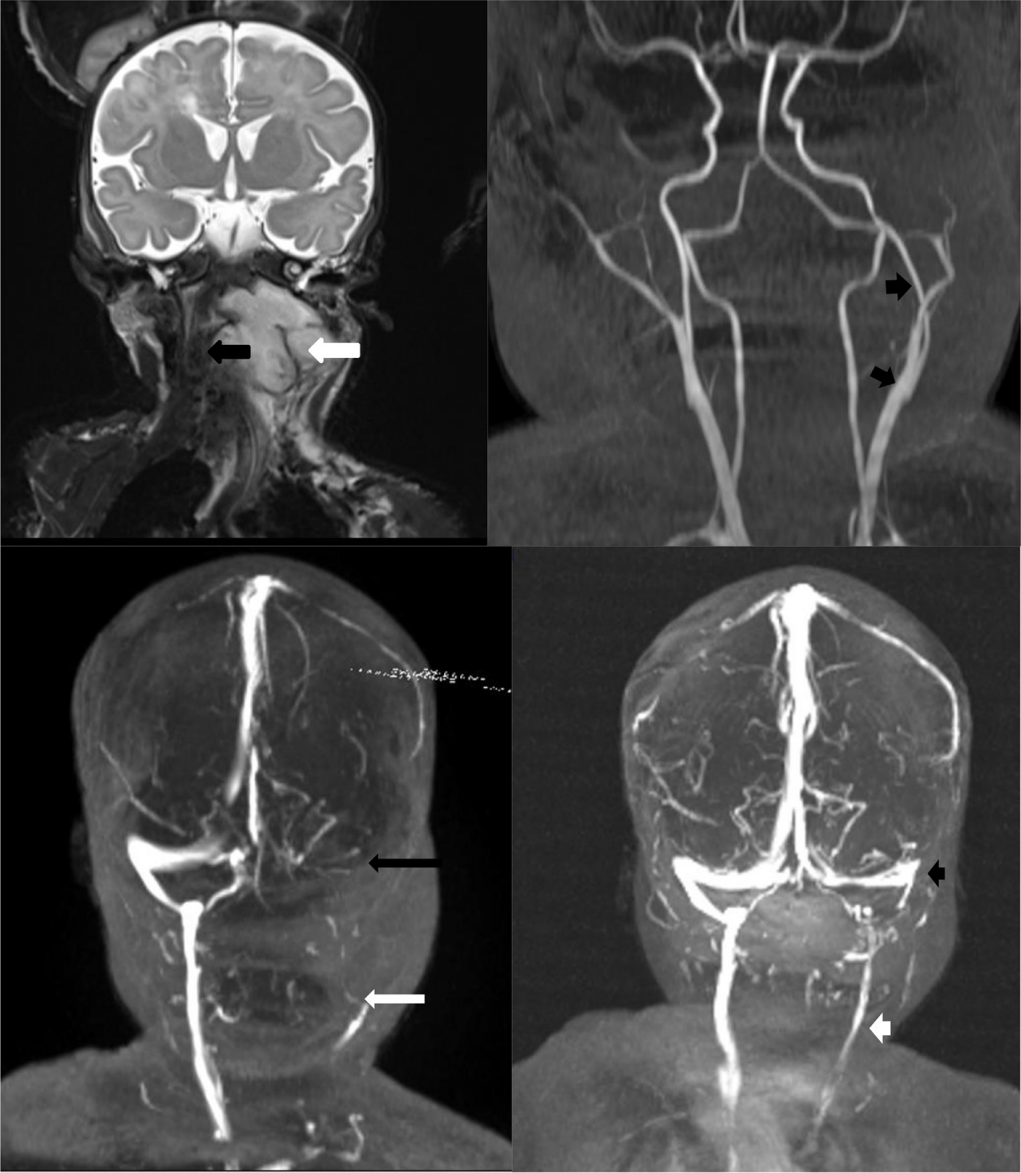
(a-d) (a) Coronal *T*_2_ weighted MR scan showing the neck abscess as a contrast-enhancing irregular lesion (white arrow), with evident tracheal displacement (black arrow showing the endotracheal tube). Note the displacement of neck arteries on the arteriogram (b) and on the venogram (c), left lateral sinus thrombosis (black arrow) seen as absence of contrast in the sinus as well as the jugular vein (white arrow). On the repeat MR venogram (d) done on the 14th day, note the recanalization of the left lateral sinus (black arrowhead) and internal jugular vein (white arrowhead).

He was further noted to have a firm, smooth, immobile left submandibular mass, extending to the neck levels II-V. A CT scan was done, revealing a 27 mm × 47 mm × 42 mm parapharyngeal/paravertebral cystic mass obliterating the oropharyngeal airway and acute left LST (Figure 1); the latter confirmed on MRI (Figure 2). He was started on low molecular weight heparin (*Enoxaparin*) at 1 mg/kg^–1^ and ultrasound-guided aspiration was done. This revealed pus and 20 ml drained and cavity flushed with 40 mg* gentamycin* and 1 g* vancomycin.Staphylococcus aureus was grown on culture of the aspirate. *There were no acid fast bacilli, and the human immunodeficiency virus (HIV) test done was negative. Ceftriaxone was then changed to clindamycin, with successful extubation 48 hours later, and transfer to the ward after 2 weeks of ICU care. A repeat MR venogram (Figure 2) done at the end of his 14 day stay in the ward showed partial re-cannulation in the sinuses.

## Discussion

Deep neck abscesses are collections of pus in the facial spaces of the head and neck. Of these, the retropharyngeal and parapharyngeal spaces are mostly affected in young children.^3^ These uncommon but potentially life-threatening abscesses are usually consequent to naso/oropharyngeal infections,^2^ with subsequent lymphadenitis, cellulitis, phlegmon and eventually abscesses.^1^ Although better imaging modalities, use of antibiotics and surgical management have led to decreased morbidity, the ease of spread of deep neck abscesses from one cavity to another can easily lead to lethal complications including airway compromise, mediastinitis, arterial erosion, bacteremia, jugular vein thrombosis and dural sinus thrombosis, especially in younger children.^2,4^ Deep neck infections can also lead to ligament laxity and spasmic contraction of cervical muscles leading to an inflammatory atlanto-axial subluxation, the syndrome of Griesel.^5^

With an estimated incidence of 0.67 per 100 000 children per year, cerebral venous sinus thrombosis is an infrequent but poorly understood condition with devastating morbidity.^6^ The most frequently thrombosed sinuses are the superior sagittal sinus and the lateral sinus, with most cases affecting both, and only a few isolated LST cases.^7^ The pathophysiology of LST (comprising the transverse and the sigmoid sinuses) in children, as in any thrombotic process, is multifactorial with risk factors associated with the classical Virchow triad including prothrombotic disorders, head trauma and infections.^6^ The latter group, septic LST, is predominant in low-resource settings and has been considered exclusively associated with otitis and mastoiditis with direct dissemination of infection and subsequent sinus wall thrombophlebitis.^8^ It is mostly a disease of adults, with few non-otogenic cases reported; these are associated with internal jugular vein (IJV) thrombosis seen in *Lemierre’s* syndrome especially in older children.^4^ There is however another overlooked, largely unidentified, group where massive local extension of infections/abscesses in the deep neck spaces can occlude the IJV, leading to retrograde stasis and thrombosis with or without direct dissemination of infection into the sinus.

The atypical etiology (non-otogenic infection), multifarious pathophysiology and nonspecific symptomatology can worsen the delay in diagnosis of such a variant of CSVT with consequent increased mortality and long-term morbidity including cognitive impairment, motor impairment and epilepsy, especially in regions with poor access to healthcare.^9,10^

Anticoagulant therapy remains controversial especially in neonates but remains the cornerstone of CSVT treatment with initial heparin and subsequent oral anticoagulation for 3–6 months, or earlier if recanalization is demonstrated on follow-up neuroimaging—preferably with MR venography—usually done at 3, 6 and 12 months after diagnosis.^10^

## Learning points

1. Most previously reported cases of LST are in adults and are otogenic in origin, arising from chronic otitis and mastoiditis. This case however illustrates a relatively rare case pertaining to isolated lateral non-otogenic sinus thrombosis in an *infant* resulting from an upper respiratory tract infection
2. While almost all cases of LST of infectious origin are considered to result from direct dissemination of infection into the lateral sinus, the complex pathophysiological mechanism is here highlighted, especially when delay in diagnosis renders neck masses massive and compressive. Knowledge of such rare cases is vital in ensuring their timely diagnosis and treatment.

## Consent

Written informed consent for the case to be published was obtained from both parents for publication of this case report, including accompanying images.
